# Prevalence of methicillin-resistant *Staphylococcus aureus* among large commercial pig herds in South Africa

**DOI:** 10.4102/ojvr.v85i1.1561

**Published:** 2018-07-17

**Authors:** Shani van Lochem, Peter N. Thompson, Cornelius H. Annandale

**Affiliations:** 1 Department of Production Animal Studies, Faculty of Veterinary Science, University of Pretoria, South Africa

## Abstract

The prevalence of nasal carrier status of methicillin-resistant *Staphylococcus aureus* (MRSA) in pigs has been described elsewhere, but is unknown in South Africa. To address concerns that exist regarding the zoonotic risk that carriers pose to workers, the herd-level prevalence of MRSA was determined among 25 large (> 500 sows) commercial pig herds in South Africa, representing 45% of the large commercial herds in the country. From each herd, the nasal contents of 18 finisher pigs were sampled at the abattoir, pooled into three and selectively cultured to determine the presence of MRSA. A herd was classified as MRSA-positive if one or more of the three pooled samples cultured positive. Three of the 25 herds tested positive for MRSA, equating to a 12% herd prevalence (95% CI: 7% – 23%) among South African commercial piggeries. The prevalence of nasal MRSA carriers among large commercial pig herds in South Africa was low compared to what has been reported elsewhere and suggests a relatively low zoonotic MRSA risk to workers in South African commercial piggeries and abattoirs.

## Introduction

Methicillin-resistant *Staphylococcus aureus* (MRSA) is one of the leading nosocomial pathogens causing hospital-associated infections in humans worldwide and is a major cause of prolonged hospital stays and mortality (Bell et al. 2002; Dantes et al. 2013; De Kraker et al. 2011; Hoyert & Xu 2012; Köck et al. 2010). Nasal carriage is one of the major risk factors for developing staphylococcal infection (Peacock, De Silva & Lowy 2001). The ability of MRSA to acquire different mechanisms of antibiotic resistance through plasmids, chromosome cassettes or both (Lowy 2003) realises its potential to be a truly multidrug-resistant pathogen.

Three MRSA epidemiological reservoirs are currently recognised: hospital-associated (HA-MRSA), community-associated (CA-MRSA) and livestock-associated (LA-MRSA) (Köck et al. 2010). Livestock-associated methicillin-resistant *Staphylococcus aureus*, carried in pigs, is seen as a potential risk for people working with pigs, as the carrier status might be transferred to them. The first human case of LA-MRSA from pigs was detected in a Dutch hospital in 2004 in a 6-month-old baby from a pig farming family (Voss et al. 2005), and LA-MRSA may account for a significant proportion of human MRSA cases, particularly CA-MRSA but also HA-MRSA (Cuny, Wieler & Witte 2015).

Prevalence and risk factor studies of LA-MRSA in both pigs and humans working with pigs have been conducted in several countries in recent years, such as the Netherlands (De Neeling et al. 2007), Germany (Alt et al. 2011), Denmark (Verhegghe et al. 2013), Italy (Normanno et al. 2015), Canada (Khanna et al. 2008) and the USA (Smith et al. 2009). An international study of the prevalence of MRSA among veterinarians (Wulf et al. 2008) estimated this to be 12.5%. In the Netherlands, individual animal MRSA prevalence among 540 pigs at nine slaughterhouses was 39% (De Neeling et al. 2007), and a subsequent study found that 67% of 171 breeding herds and 71% of 31 finisher herds were MRSA-positive (Broens et al. 2011). In contrast, reports of low MRSA-LA prevalence in pigs have been published in Nigeria (Okunlola & Ayandele 2015) and the USA (Sun et al. 2015).

In South Africa, the prevalence of LA-MRSA in pigs and people working with pigs is unknown. Introducing LA-MRSA into human hospitals is potentially dangerous in a country with a high prevalence of HIV infection, which is one of the risk factors for acquiring an MRSA infection (Shisana 2005). Therefore, in order to better understand the risk posed to pig caretakers and abattoir workers, this study aimed to determine the herd prevalence of LA-MRSA among commercial pig herds in South Africa.

## Materials and Methods

### Study design

A cross-sectional survey with two-stage sampling was used. A random sample of large commercial pig herds of over 500 sow units was selected to represent the country’s commercial pig sector. From each herd, nasal swab samples were taken at the abattoir, then pooled and cultured to determine the herd’s MRSA infection status.

### Sample size calculation

The number of herds needed to estimate an expected prevalence (*P*_*exp*_) of 50% and a desired absolute precision (*d*) of 15% was calculated using the following equation (Thrusfield 2005):

$$n=\frac{1.96^{2}P_{exp}(1-P_{exp})}{d^{2}},$$ [Eqn 1]

giving a required sample size of 43 herds. However, as the population (*N*) of herds with ≥ 500 sow units in South Africa was only 56 herds, and the calculated sample size (*n*) was larger than 0.1*N, an adjusted sample size (*n*_*adj*_) was calculated as follows (Thrusfield 2005):

$$n_{adj}=\frac{n\times N}{N+n}$$ [Eqn 2]

Therefore, 25 herds were selected for this study. The number of pigs required to sample in order to detect the presence of MRSA in a herd using the weekly batches of pigs sent to the abattoir was calculated using FreeCalc2 software (www.ausvet.com.au). A minimum expected (design) prevalence of 20% was used, with a test sensitivity of 80%, specificity of 100%, alpha = 0.05 (significance level) and beta = 0.05 (1 – power). The sample size calculated to detect colonisation among the largest expected batch (2500 pigs from a 5000 sow-unit) was 18 pigs.

### Sample collection

The nasal contents of 18 finisher pigs from each participating herd were sampled at the abattoir between stunning and exsanguination. A single sterile swab (Copan Transystem^®^, Copan Diagnostics Inc., Murrieta, USA) was used to collect the contents of both nares of each selected pig.

### Laboratory procedures

The 18 samples from each herd were pooled into three pools of six swabs per pool. The pooled samples were directly swabbed onto chromID^™^ MRSA agar plates (bioMérieux SA, Marcy-l’Etoile, France) and used to screen for MRSA (Graveland et al. 2009). With each batch of chromID^™^ MRSA agar plates processed, one plate was inoculated with a known MRSA-positive strain as a positive control. Plates were pre-tested at the manufacturer using ATCC 43300 as a positive control and ATCC 29213 as a negative control. These plates were incubated in aerobic conditions at 37 °C for 48 hours.

Positive plates were selected for a rapid slide agglutination test (Staphaureux*, Remel Europe Ltd, Kent, UK) (Moser et al. 2013) to confirm whether the colonies were truly *S. aureus*. An authentic MRSA strain was used as a positive control for each batch of suspected colonies tested with Staphaureux*.

If a sample was positive both on the chromID^™^ MRSA agar plate and on the Staphaurex* slide, colonies were then sampled from the MRSA agar plate for further confirmation by mass spectrometry. A pooled sample was concluded to be MRSA-positive with 100% specificity only if it was identified as *S. aureus* on mass spectrometry. Resistance to cefoxitin, using the cefoxitin disk diffusion test, was confirmed in a random selection of five positive samples with an antibiogram; all five samples were resistant.

To investigate the possible effect of pooling on the sensitivity of the culture method, 24 swabs from four herds, of which two tested positive and two negative, were individually inoculated directly onto the chromID^™^ MRSA agar plates. To ensure that the absence of a pre-enrichment broth was not affecting results, samples were individually enriched with thioglycollate broth for 24 h at 37 °C, after which an inoculum was then inoculated onto the chromID^™^ MRSA agar plate, which was read after 24 h. Lastly, to rule out the possibility that Gram-negative bacteria were inhibiting the growth of MRSA, individual swabs were used to inoculate 5% sheep blood agar swabs containing colistin for 24 h at 37 °C, whereafter the colonies were inoculated onto the chromID^™^ MRSA agar plates.

### Data analysis

A herd was classified as MRSA-positive if at least one pooled nasal sample tested positive for MRSA. The estimated MRSA herd prevalence in the finite population of 56 large commercial herds was calculated with exact hypergeometric 95% confidence intervals using StatCalc 2.0 (Krishnamoorthy 2006).

## Results

Of the 75 pooled samples, representing 450 pigs from the 25 participating herds, seven pooled samples from three herds were positive on both the selective chromID^™^ agar plate and the Staphaureux* agglutination test. All positive colonies were identified to be *S. aureus* on mass spectrometry. All positive colonies tested on an antibiogram were resistant to cefoxitin. Methicillin-resistant *Staphylococcus aureus* herd prevalence among large commercial piggeries in South Africa was therefore estimated to be 12% (95% CI: 7% – 23%).

From the 24 individual swabs from four herds, all 12 swabs from the two negative herds tested negative, while in the two positive herds, all six individual swabs from one herd and three individual swabs from the other herd tested positive on the chromID^™^ MRSA agar plates. All 12 swabs from the negative herds tested negative after pre-enrichment, while all six swabs from one positive herd and three swabs from the other positive herd tested positive after pre-enrichment. After pre-inoculation onto 5% sheep blood agar plates containing colistin, the swabs from the two negative herds all tested negative on the MRSA selective plates, while all six swab samples from the one positive herd and three swabs from the other positive herd tested positive on the MRSA plates.

### Ethical considerations

This study was approved by the Animal Ethics Committee of the University of Pretoria (V093/14).

## Discussion and conclusion

This study estimated the herd prevalence of MRSA in large commercial piggeries in South Africa to be between 7% and 23%, with 95% confidence. Despite the relatively large range of the estimate, it can be concluded that less than one quarter of large piggeries in South Africa are likely to have MRSA carriers. This is much lower than reported by Broens et al. (2011) in the Netherlands, where herd prevalences of 67% in breeding herds and 71% in finisher herds were observed; and by Alt et al. (2011) in Germany, where 52% of fattening pig farms tested positive for MRSA. However, in both Nigeria and the USA, low MRSA herd prevalences have been described. In Ilora, Nigeria, MRSA herd prevalence from 11 participating herds was 9% (Okunlola & Ayandele 2015), consistent with the 12% herd prevalence found in South Africa in this study. A recent study in the USA found no MRSA in 36 herds across 11 states (Sun et al. 2015). It may be that the low MRSA herd prevalence in these three countries is related to the lower pig density than in countries such as the Netherlands. Increased distances between piggeries may increase overall biosecurity, resulting in less spread of disease between herds, with consequently healthier herds and an overall reduction in the use of antimicrobial and other drugs. The possible influence of pig density on MRSA herd prevalence warrants further investigation.

As the occurrence of LA-MRSA is reported to be strongly associated with herd size, occurring more frequently in medium and large herds (Alt et al. 2011), this study investigated the sector of the South African pig population that was likely to have the highest prevalence of LA-MRSA. The prevalence among smaller and non-commercial pig farms is therefore likely to be even lower than in this study. Although MRSA has been reported in pigs from small-scale farms in the Eastern Cape province of South Africa (Adegoke & Okoh 2014), its prevalence in such farming systems has not been reported and requires further investigation.

It is possible that the prevalence of MRSA in infected herds may have been underestimated if the herd-level sensitivity of the test was low; this would have been the case if the specified design prevalence of 20% was too high. However, it was far less when compared to the 75% within-herd prevalence reported in finisher pigs (Verhegghe et al. 2013) and the 39% prevalence in pigs at slaughterhouses (De Neeling et al. 2007) in the Netherlands. The true within-herd prevalence in South African pig herds and the age of pigs with the highest MRSA prevalence require further investigation.

Herds were only sampled once and not repeatedly, whereas both the herd and within-herd prevalence of MRSA may change over time. The risk of herds acquiring MRSA is not only dependant on transmission between carriers and non-carriers, but is significantly increased via the administration of antimicrobials to groups of pigs (Van Duijkeren et al. 2008). Group antimicrobial administration might change periodically within a herd, depending on the treatment programme followed on the farm prior to sampling. It could also be seasonal if one considers an increase in severity of respiratory diseases during autumn and winter. The influence of antimicrobial usage patterns was not investigated in this study.

Our results indicate that LA-MRSA in pigs in South Africa is likely of limited significance as a public health threat to workers on farms and abattoirs. Further research should focus on quantifying the true public health risk through investigating possible temporal or seasonal variations in prevalence, estimating within-herd MRSA prevalence in pigs and establishing the LA-MRSA carrier status of personnel working with pigs on farms and in abattoirs.
